# Exceptional soft tissue preservation reveals a cnidarian affinity for a Cambrian phosphatic tubicolous enigma

**DOI:** 10.1098/rspb.2022.1623

**Published:** 2022-11-09

**Authors:** Guangxu Zhang, Luke A. Parry, Jakob Vinther, Xiaoya Ma

**Affiliations:** ^1^ Yunnan Key Laboratory for Palaeobiology and MEC International Joint Laboratory for Palaeobiology and Palaeoenvironment, Institute of Palaeontology, Yunnan University, Kunming, People's Republic of China; ^2^ Department of Earth Sciences, University of Oxford, Oxford, UK; ^3^ Schools of Earth Sciences and Biological Sciences, University of Bristol, Bristol, UK; ^4^ Centre for Ecology and Conservation, University of Exeter, Penryn, UK

**Keywords:** Cambrian, Cnidaria, Medusozoa

## Abstract

Exoskeletal dwelling tubes are widespread among extant animals and early fossil assemblages. Exceptional fossils from the Cambrian reveal independent origins of tube dwelling by several clades including cnidarians, lophophorates, annelids, scalidophorans, panarthropods and ambulacrarians. However, most fossil tubes lack preservation of soft parts, making it difficult to understand their affinities and evolutionary significance. *Gangtoucunia aspera* (Wulongqing Formation, Cambrian Stage 4) was an annulated, gradually expanding phosphatic tube, with occasional attachments of multiple, smaller juveniles and has previously been interpreted as the dwelling tube of a ‘worm’ (e.g. a scalidophoran), lophophorate or problematicum. Here, we report the first soft tissues from *Gangtoucunia* that reveal a smooth body with circumoral tentacles and a blind, spacious gut that is partitioned by septa. This is consistent with cnidarian polyps and phylogenetic analysis resolves *Gangtoucunia* as a total group medusozoan. The tube of *Gangtoucunia* is phenotypically similar to problematic annulated tubular fossils (e.g. *Sphenothallus*, *Byronia*, hyolithelminths), which have been compared to both cnidarians and annelids, and are among the oldest assemblages of skeletal fossils. The cnidarian characters of *G. aspera* suggest that these early tubular taxa are best interpreted as cnidarians rather than sessile bilaterians in the absence of contrary soft tissue evidence.

## 1. Introduction

A tubicolous mode of life is common among animals, with the tubular exoskeleton providing a variety of functions including protection from predation, supporting and elevating the body during feeding, assisting in respiration in oxygen poor environments (e.g. within sediments) and isolating animals from hostile surrounding conditions (e.g. polychaetes at hydrothermal vents) [1]. Tubes are common among the oldest assemblages of skeletal fossils in the latest Ediacaran and early Cambrian [2,3], indicating the importance of this lifestyle during the assembly of the oldest animal-dominated communities. Examples of early tube builders are known from across the animal tree of life, including cnidarians [4], annelids [5], hemichordates [6], lobopodians [7] and scalidophorans [8] as well as numerous examples with unknown or controversial phylogenetic affinities [3,9,10].

Given their morphological simplicity, fossil tubes are oftentimes difficult to diagnose with confidence to a particular taxon, and most early fossil tubes lack preservation of soft tissues. This uncertainty obfuscates the evolutionary and ecological significance of many of the oldest skeletal fossils. The tubes of extinct animals do not necessarily closely resemble those of extant analogues (e.g. agglutinated tubes in the extant priapulid *Maccabeus* [11] versus annulated, cuticular tubes in the fossil scalidophoran *Selkirkia* [8]), and direct comparison between tube morphology of living and extinct species may, therefore, not provide sufficient evidence to diagnose fossil tubes in the absence of associated soft tissues.

The controversial and uncertain identity of fossil tubes is exemplified by the late Ediacaran fossil *Cloudina* and relatives, which has been repeatedly interpreted and re-interpreted as an annelid or cnidarian [3,12]. Similar annulated tubes are common components of earliest Cambrian skeletal assemblages [2] and have diverse morphologies and construction materials. These include a suite of fossils that reveals a diversity of early cnidarians, such as early developmental stages of extinct medusozoans and ‘protoconulariids’, tubes with a polygonal cross section with close similarities to co-eval cnidarians [13] and younger Palaeozoic conulariids [14]. The affinities of the phosphatic hyolithelminths are somewhat more mysterious and they are compared to both annelids and cnidarians [15,16], as with cloudinimorphs [3,10]. However, they also closely resemble the organo-phosphatic tubes of *Sphenothallus*, *Byronia* and *Tubulella*, more often interpreted as cnidarians [15,17]. Soft tissues from these genera are almost completely unknown, with the only example from the Devonian Hunsrück Slate, where three-dimensional pyritization reveals a bilaterally symmetrical tentacle apparatus [18]. While this specimen was originally interpreted as annelid-like these features can also be accommodated by a cnidarian interpretation [19].

In the light of this lack of soft tissue evidence, these tubicolous taxa have also been interpreted as annelids, specifically siboglinids [20], a group with which many early annulated tubular fossils are often compared [3,9], despite a lack of unambiguous evidence for this group and their close relatives not appearing until much later in the fossil record [21]. In general there is a lack of preservation of soft tissues associated with putative cnidarians in Cambrian Lagerstätten, and some authors have advocated extreme caution in assigning these organisms at phylum level due to the absence of diagnostic features [22]. However, given that these taxa are stratigraphically long lived and geographically widespread, our understanding of their role in Palaeozoic ecosystems is hampered by a lack of clear understanding of their affinities.

**Figure 1.**
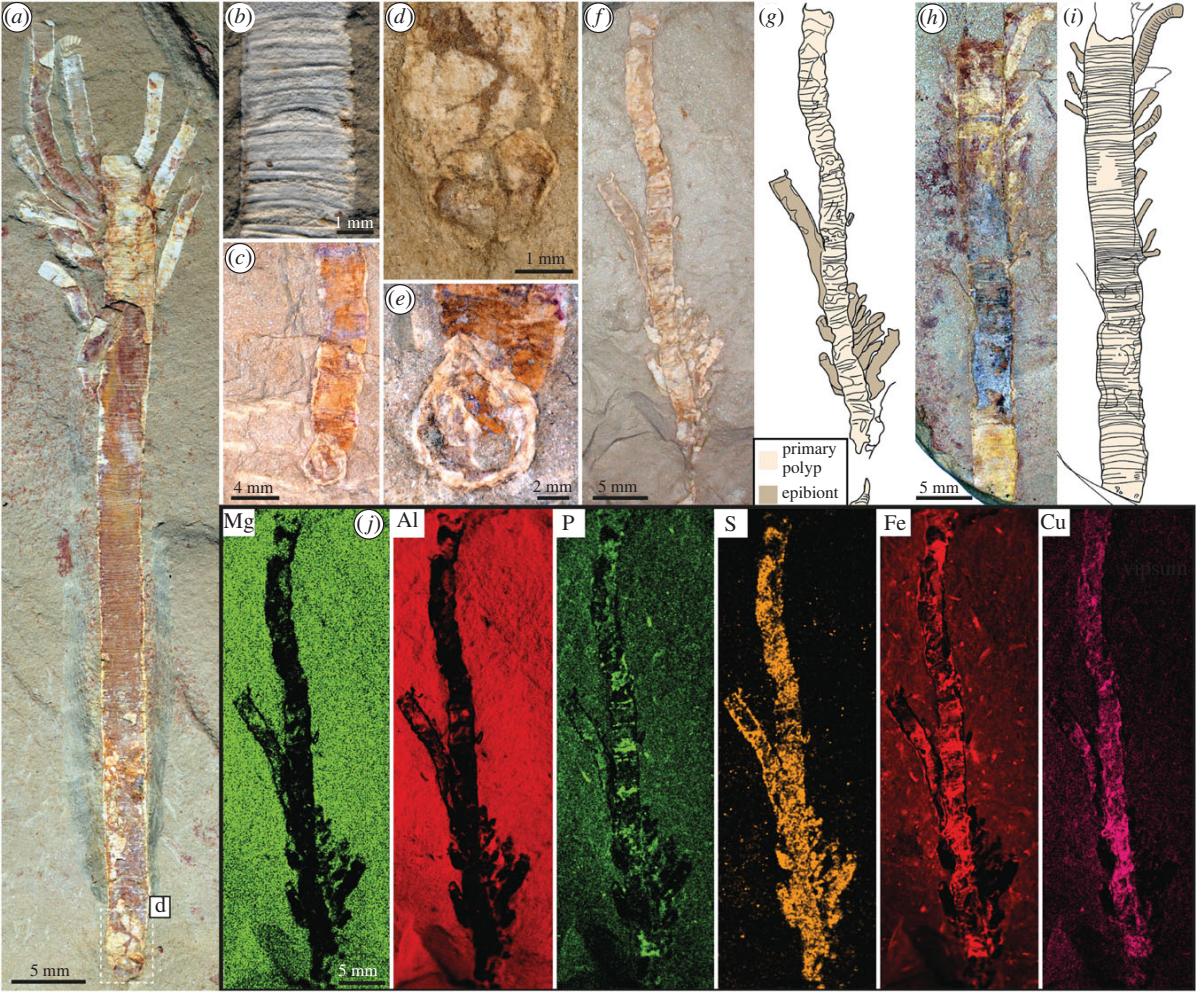
Tube morphology, composition and epibionts in *Gangtoucunia aspera.* (*a*,*d*) YKLP 11439, complete adult tube with conspecific epibionts and preserving the apical attachment structure. (*b*) YKLP 11436, showing detail of transverse tube ornament. (*c*,*e*) Specimen kg-f-2-10, with apical attachment structure/holdfast. (*d*) Detail of apical attachment structure in YKLP 11439. (*e*) Detail of apical attachment structure of specimen kg-f-2-10. (*f*,*g*) YKLP 11435, with conspecific epibionts (*f*) and interpretative drawing (*g*). (*h*,*i*) YKLP 11434, adult tube with small conspecific epibionts (*h*) and interpretative drawing (*i*). (*j*) Elemental mapping of YKLP 11435. (Online version in colour.)

**Figure 2.**
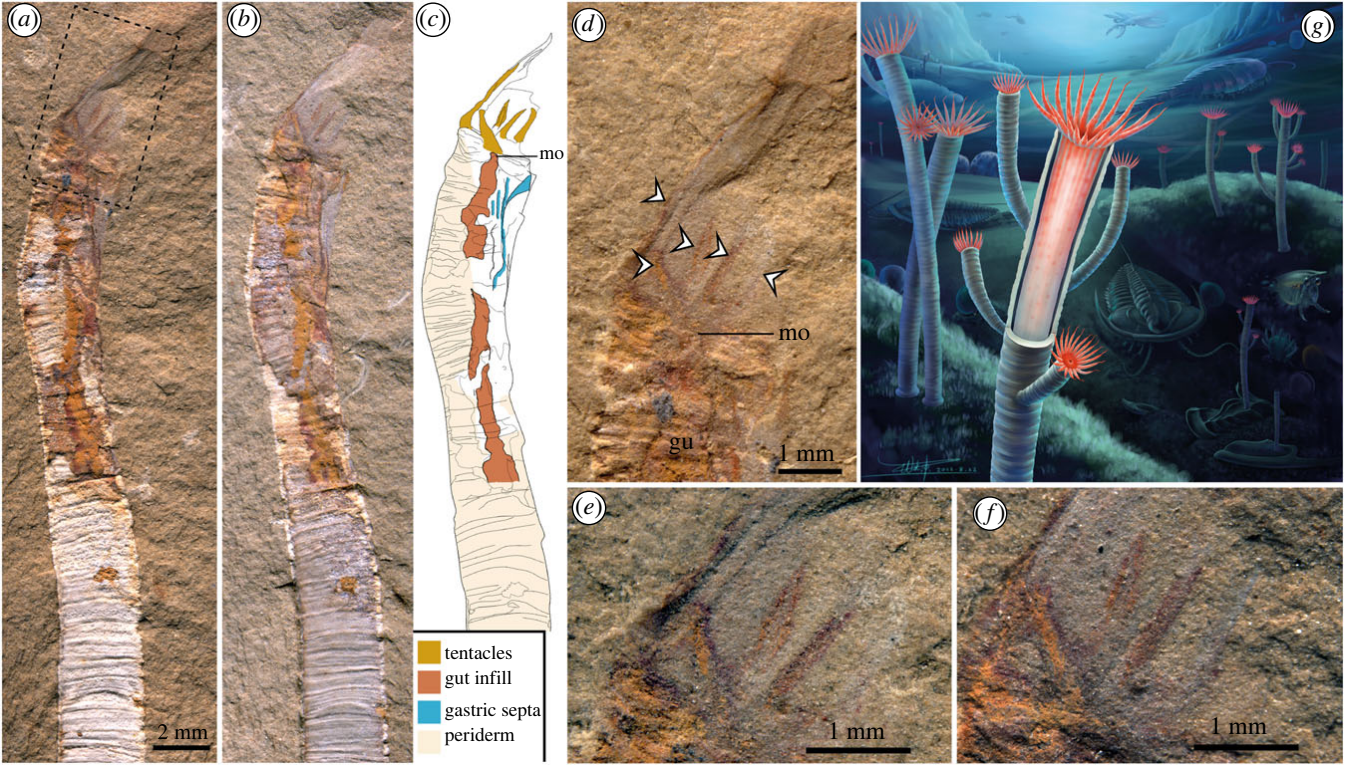
Specimen YKLP11436 preserved *in situ* in a dwelling tube with soft tissues and life reconstruction. (*a*) Overview of oralmost region of part, boxed region shown in (*d*). (*b*) Overview of oralmost region of counterpart. (*c*) Interpretative drawing combining information from both part and counterpart. (*d*) Detail of oral region and tentacular apparatus of part. (*e*) Close up of tentacles in part. (*f*) Close up of tentacles in counterpart. (*g*) Life reconstruction of *G. aspera* by Xiaodong Wang. (Online version in colour.)

**Figure 3.**
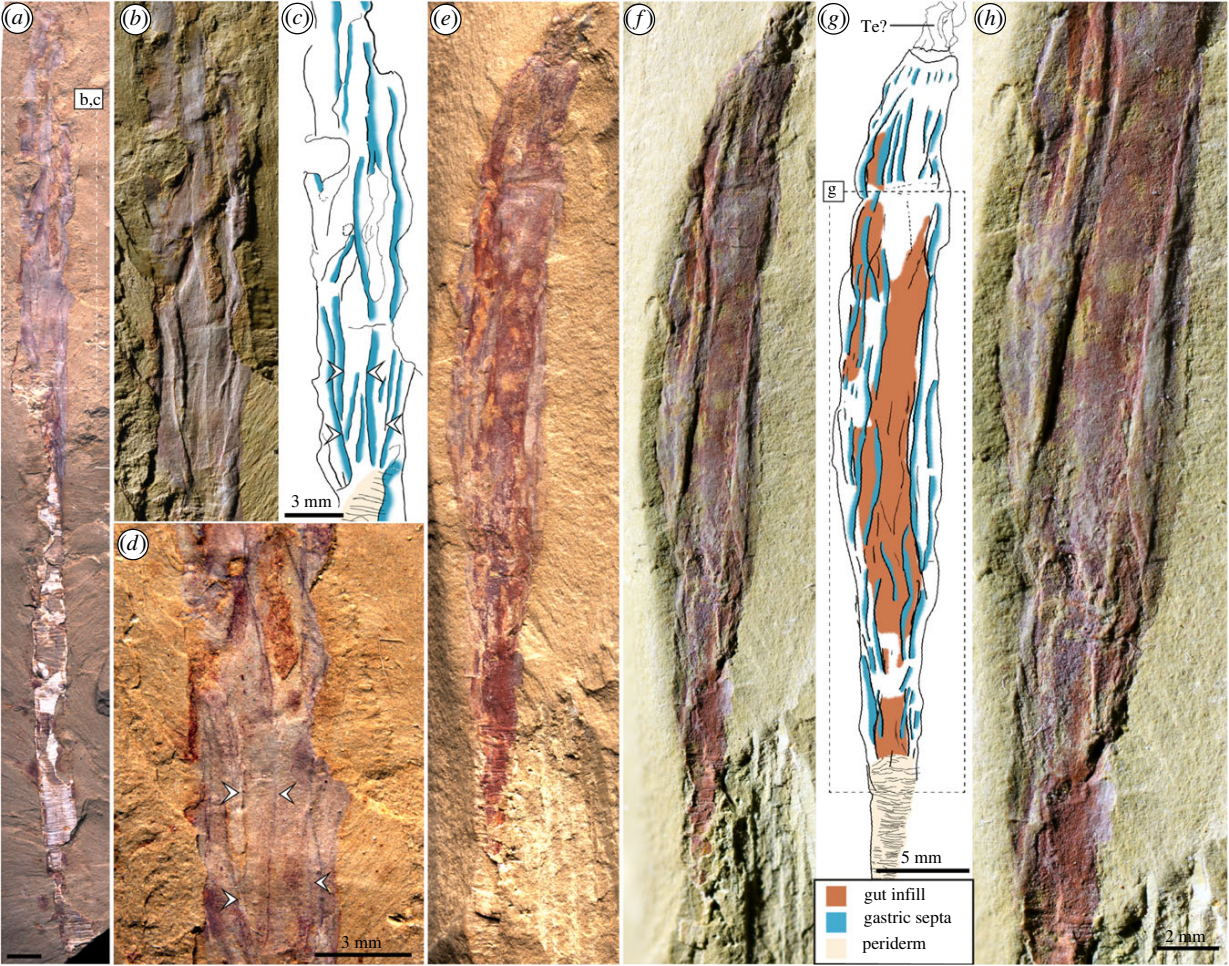
Specimens preserving internal details of the digestive system. (*a*–*d*) YKLP 11437; (*e*–*h*) YKLP 11438. (*a*) Overview of specimen showing soft tissues partially extruded from the dwelling tube. (*b*) Detail of boxed region in (*a*) showing longitudinal gastric septa using low angle lighting. (*c*) Interpretative drawing of (*b*) highlighting the morphology of the digestive tract and gastric septa. (*d*) High angle light image of digestive tract showing extent of the gastric septa demarcated by dark linear structures. (*e*) Overview of specimen with high angle illumination. (*f*) Overview of specimen as in (*e*) with low angle illumination. (*g*) Interpretative drawing of region shown in (*e*,*f*) highlighting the trajectory and morphology of the digestive tract. (*h*) Close up of three dimensionally preserved septa. (Online version in colour.)

*Gangtoucunia aspera* is a problematic, annulated, tubular and phosphatic fossil from the Guanshan Lagerstätte (Cambrian Stage 4) from eastern Yunnan Province, China, which is broadly morphologically similar to hyolithelminths and *Byronia*. The affinities of *Gangtoucunia* have been little explored since it was described, but it has been interpreted as some kind of ‘worm’ among scalidophorans (see [23] and electronic supplementary material, table S1 [24]), as a member of Lophophorata [25] or a problematic taxon [26]. Here, we describe new specimens of *G. aspera*, which reveal its originally phosphatic tube (figure 1) and exceptionally preserved internal soft tissues for the first time (figures 2 and 3), shedding new light on its phylogenetic position and evolutionary significance.

## 2. Material and methods

The specimens were collected from the Gaoloufang section at Guangwei village, Kunming city, eastern Yunnan Province, China. This site belongs to the lower part of the Wulongqing Formation, *Palaeolenus lantenoisi* zone, Cambrian Stage 4. All specimens are housed at the Yunnan Key Laboratory for Palaeobiology, Yunnan University, Kunming, China. Seventy-six specimens were analysed in total, including eight specimens that formed a substrate for multiple juveniles to attach to, two with a holdfast, and four with soft tissue associated with the tube.

Specimens were photographed with a Keyence VHX 6000 digital microscope and a Nikon D850 DSLR camera. Elemental maps were obtained using a Bruker M4 Tornado micro X-ray fluorescence (XRF) spectrometer instrument. All analyses were conducted under vacuum pressure of 2.4 mbar, X-ray tube voltage of 50 kV and currents of 599 µA with no primary beam filters.

Phylogenetic and tube morphospace analyses were carried out using modified datasets from Dunn *et al.* [27], which incorporated *G. aspera* along with several minor changes and additions (see electronic supplementary material)*.* Phylogenetic analyses were run in MrBayes v.3.2.7 using the Mkv model with a gamma distribution for character rate variation; 100 000 000 generations were requested, with the analyses terminating once the average standard deviation of split frequencies dropped below 0.01. The analysis reached convergence after 5 695 000 generations. Convergence was assessed using the effective sample size (ESS) and potential scale reduction factor (PSRF) scores calculated using the sump command and 25% of the sample was discarded as burn-in.

Tube morphospace analyses were performed using R, following a similar approach to previous analyses [27]. Gower distances were computed using the package *daisy*, and non-metric multidimensional scaling with four axes was performed using *vegan* (stress less than 0.1). Minor modifications have been made to the previous iteration of the tube morphospace dataset. These include the addition of the tubicolous priapulid *Selkirkia* [8] and the expansion of the taxon sample to encompass multiple species of *Hyolithellus* and *Byronia*, as previous character scores did not capture some of the morphological variations in these taxa. *Hyolithellus insolitus* was added as it has a longitudinal ornament similar to many *Byronia* species [28] and *Hyolithellus* cf. *micans* is scored for the taxon known in life position from Greenland [15]. *Byronia annulata* is rescored as uncertain for the presence of biomineralization [15] and *Byronia jaegeri* [29] is incorporated as it possesses a similar irregular transverse annulation to *G. aspera*, which is scored as an additional character not present in the original dataset.

## 3. Results

**Systematic palaeontology**

Cnidaria Verrill, 1865 nov.

Medusozoa Peterson, 1979 nov.

*Gangtoucunia aspera* Luo & Hu, 1999

**Emended diagnosis:** Elongated, tubular polypoid cnidarian that secreted an organophosphatic dwelling tube. The external surface of the tube is transversely annulated with dense and irregular growth lines and has a basal, disc-shaped holdfast. The tube is elongate with a slight, gradual proximal tapering. The polyp has a single whorl of circumoral tentacles of unknown total length. The polyp body is externally smooth. Internally, the gut is divided by numerous longitudinal septa that partition an elongate gut extending along the preserved length of the body.

**Description:** The tubes of *Gangtoucunia* are approximately straight with some irregular, gentle curvature (figures 1 and 2*a–c*). Towards the aboral region the tube tapers gently, with tapering almost absent in the remaining part. Two specimens preserve an apical structure that is consistent with an attachment structure/holdfast (figure 1*a*,*c–e*), suggesting that the tube was attached to a substrate in life and open only at one end. This is also consistent with the observation of multiple smaller tubes attached to large tubes, suggesting conspecific epibionts (figure 1*a*,*f*,*h*). *Gangtoucunia* tubes or morphologically similar holdfast structures are also observed encrusting relatively robust bilaterian carcasses, including non-biomineralized arthropods (electronic supplementary material, figure S1a,b), trilobites (electronic supplementary material, figure S1c,d) and vetulicolians (electronic supplementary material, figure S1e,f), suggesting a general preference for attachment to hard substrates.

The tube is densely and irregularly annulated, with approximately 3–10 transverse annulations per millimetre and more prominent annulation every approximately 10th annulation. The annulations are irregular in width (figure 1*b*) and approximately straight, suggesting that the tube was circular (or sub-circular) prior to compaction. There is no obvious longitudinal ornament (i.e. that is characteristic of the type species of *Byronia* and some taxa currently referred to as *Hyolithellus* [15]).

The skeleton is preserved in high relief as an off-white material with an associated rusty coating. The high relief preservation is consistent with an originally biomineralized composition that was presumably calcium phosphate, which is further indicated by the enrichment of phosphorus relative to the matrix in well-preserved regions of the tubes. Due to the compacted and flattened nature of the fossils, internal features such as cusps or peridermal teeth (e.g. as in coronates and visible in some fossil species, e.g. *Olivooides* [30] and *Sphenothallus* [31]) cannot be observed.

Soft tissues are preserved as dark and rust-coloured films and were observed in four specimens. In two specimens the soft tissues emerge directly from the tube aperture, whereas in the others the distal portion of the soft anatomy is displaced up to 37.3 mm from the end of the tube, suggesting partial extrusion and effacement of the animal during or immediately prior to burial.

In YKLP 11436 (figure 2), the body is preserved *in situ* inside the tube, with tentacles emerging from the body close to the position of the tube aperture. The tentacles are 4.7 mm long where fully exposed and approximately 2.5 mm wide. They are preserved with the greatest fidelity proximally where they are clearly differentiated as orange and dark coloured remains with some relief. The tentacles are smooth, unbranched and gently tapering. A total of five tentacles can be clearly identified but there is a region of non-preservation to the right-hand side of the part, indicating more tentacles would have been present along the width of the specimen. Given that the specimen is preserved in lateral view and only a subset of the tentacles is visible (i.e. as in other polypoid fossils like dinomischiids [32]), the total number of tentacles was well in excess of six in life, totalling approximately 18.6, given a circular outline and calculating the circumference by the preserved diameter divided by the tentacle width of the tentacle exposed medially on this specimen.

The tentacles encircle the mouth, which is connected to a long, tubular gut, that is preserved as a wide, rust-coloured impression with low relief in YKLP 11437 figure 3*a*–*d* and YKLP 11438 figure 3*e*–*h*. Longitudinal divisions of the gut can be seen at the oral end of the tentacle bearing specimen (YKLP 11436), where the skeleton has been removed during splitting. These partitions (gastric septa hereafter) are revealed as longitudinal dark lines when illuminated with high angle light, but are also visible as relief structures in low angle light, with breaks of slope indicating the position of overlapping individual gastric septa. This three-dimensional morphology indicates that these features are sheets of tissue that projected from the wall of the gut into the lumen, partitioning the gut longitudinally. Preservation of these septa is similar to that described in early Cambrian ‘dinomischiids’ where the guts have not been infilled (e.g. Figure 1*g* in [32]). As with the tentacles, the number of mesenteries is difficult to discern with confidence, but assuming a circular body, an equal spacing of septa and dividing the best exposed spacing between the mesenteries with the calculated circumference yields a number between 15.96 and 19.1 mesenteries, which is potentially indicative of a one-to-one correspondence between the tentacles and mesenteries. The same method has previously been used to calculate the number of gastric septa and tentacles in dinomischiids [32]. The mesenteries can be seen along the entire visible length of the body, but are obscured by the tube in the most aboral region of the specimen preserving most of its length.

**Figure 4.**
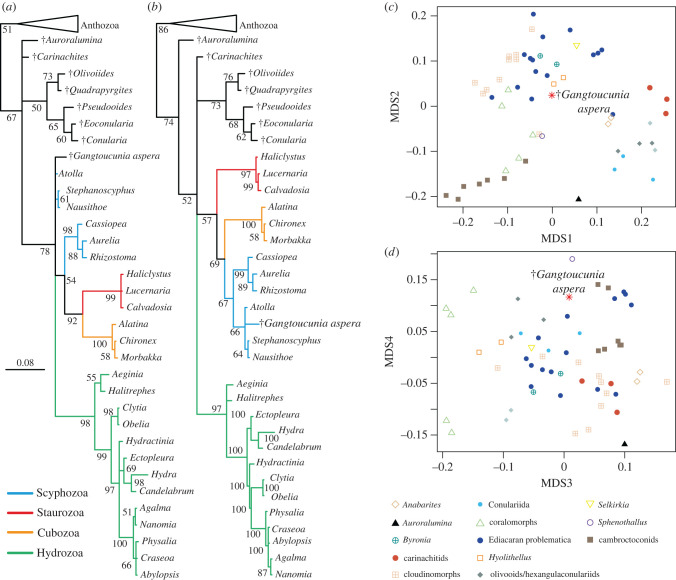
Summary of phylogenetic analyses and morphospace of the Ediacaran–Cambrian tubicolous taxa. (*a*) Analysis without topological constraint, scale bar and branch lengths in units of expected numbers of substitutions per site, numbers at nodes are posterior probabilities expressed as percentages. (*b*) Analysis with topological constraints on cnidarian relationships. (*c*) Scatterplot of NMDS axes 1 and 2. (*d*) Scatterplot of NMDS axes 3 and 4. (Online version in colour.)

Our phylogenetic analyses recover *Gangtoucunia* as a medusozoan, either in a polytomy with extant taxa (figure 4*a*) or within the scyphozoan crown group (figure 4*b*) with or without topological constraints, respectively. Analyses of tube morphospace characters find the smallest pairwise distance between *G. aspera* and *Hyolithellus micans* and is positioned proximally to hyolithellids, byroniids and problematic, annulated terminal Ediacaran taxa (figure 4*c,d*).

## 4. Discussion

Soft tissue preservation is rare among organisms enclosed by hard skeletons. The fortuitous association of soft bodies of *Gangtoucunia* partially within their phosphatic dwelling tubes offers the means to distinguish between competing hypotheses for the affinities of *Gangtouconia* and that of similar tubes that are known from skeletal fossil assemblages. The presence of a smooth external body surface with a thin body wall that enclosed a large digestive cavity partitioned by longitudinal septa and a crown of circumoral tentacles occurs only among polypoid cnidarians. The body plan observed in *Gangtoucunia* excludes previously proposed affinities for *Gangtoucunia* and (potentially) other morphologically similar tubes (e.g. scalidophorans and polychaete annelids). Externally annulated skeletons with accretionary growth are characteristic of some living medusozoans and similarly enclose the body in extant coronate scyphozoans [33]. However, such an extensive skeleton is widespread in the early evolutionary history of medusozoan cnidarians, occurring in taxa that deviate from the character combinations of extant scyphozoans in terms of body symmetry, the presence of direct development and tube biomineralization [13,30,34]. We, therefore, reconstruct *G. aspera* as a benthic medusozoan polyp, that was attached to hard substrates in life (figure 2*g*).

While some of these soft tissue characteristics (circumoral tentacles, radial symmetry, a septate blind gut) and skeletonization are also present in dinomischiids (potential stem group ctenophores [30]), dinomischiid hard parts appear to be endoskeletal and are in close association with particular anatomical features (i.e. supporting the tentacles and calyx) [32], rather than enclosing the body as in *Gangtoucunia*. None of the proposed apomorphies linking dinomischiids and ctenophores are present in *Gangtoucunia.* The number of gastric septa in *Gangtoucunia* inferred above (approx. 16–19) greatly exceeds that observed in the polyps of crown group medusozoans, where only four are present [35]. Regardless of whether the number of mesenteries/tentacles calculated is an overestimate impacted by taphonomic distortion and collapse of the body, there are at least seven visible in lateral view provided by the studied material (figure 3) suggesting that the number in *Gangtoucunia* substantially exceeded that present in the extant medusozoan lineages and is instead comparable to the condition in Anthozoa. Other taxa known from the Cambrian suggest that many gastric septa are the likely plesiomorphic condition for the cnidarian total group, with approximately 28 such septa present in the stem medusozoan *Conicula striata* [36] and 18 gastric septa in dinomischiids [32]. Regardless of competing interpretations of the latter as either stem ctenophores [32] or stem cnidarians [37,38] these taxa lie outside of the cnidarian crown group, and likewise suggest that in excess of 10 septa is plesiomorphic, with the four present in crown medusozoans representing a reduction.

Numerous similar tubicolous taxa with annulated external morphologies and accretionary growth span the Ediacaran–Cambrian boundary and have been the subject of numerous competing interpretations. These tubes vary in their composition, including groups that are organic (e.g. *Sabellidites* and *Byronia*), phosphatic (e.g. *Hyolithellus*, *Sphenothallus* and conulariids and related taxa) or calcareous (e.g. *Cloudina* and *Anabarites*). These tubes have all previously been interpreted as cnidarians based on their overall morphology and skeletal microstructure/composition and rare instances of soft tissue preservation, such as ephyrae [30]. Soft tissues from early Palaeozoic adult tubes are almost entirely unknown, with the exception of Devonian *Sphenothallus* [18]. *Sphenothallus* tubes from the Ordovician exhibit longitudinal projections of the tube wall that are comparable to peridermal teeth [31], which has been used as evidence for a polypoid body plan with a gut partitioned by septa. Cnidarian affinities are confidently known for conulariids and related taxa due to the presence of a periderm like exoskeleton with projections resembling the peridermal teeth of extant scyphozoans [39,40]. Recently, anabaritids have more confidently been placed with cnidarians based on the discovery of well-preserved endocasts that demonstrate the presence of a lobate oral aperture, a feature that is present in several groups of extinct cnidarians, including olivooids, carinachitids and conulariids [13]. Attachment to hard substrates has been demonstrated in several of these groups, including conulariids and *Sphenothallus* [41]. Similar conspecific epibiosis has been demonstrated in the conulariid *Eoconularia* [42]. Tubes with a similar mode of preservation to *Gangtoucunia* encrusting brachiopods have been described from the Guanshan Biota [43]. While these tubes likely had a similar biomineralized composition and accretionary growth [43], they lack the characteristic and prominent irregular transverse ornament shown in *Gangtoucunia*.

The tube of *Gangtoucunia* exhibits similarities with a number of taxa including possession of phosphatic mineralogy (*Spenothallus*, conulariids, hyolithelminths), irregular transverse annulation and lack of longitudinal ornament (certain *Byronia* species, e.g. *B. jaeggeri*, *Sabellidites*, *Sinotublites*) and an apical attachment structure. Although most of these taxa have been interpreted as annelids previously, the substantial overlap in characters among these tubes and the high morphological variability of taxa with clear cnidarian affinities (e.g. biomineralogy, ultrastructure, external ornament) we argue is more consistent with a highly diverse radiation of early cnidarians. While *Hyolithellus* has previously been interpreted as a *Chaetoptreus*-like annelid [15], our tube morphospace analysis demonstrates close morphological similarity between the tubes of *Gangtoucunia*, *Byronia* and *Hyolithellus,* consistent with a close relationship and shared affinity among these taxa (figure 4*c*,*d*). Nevertheless, taxonomic/morpho-groups are dispersed in our NMDS spaces (figure 4*c*,*d*), in overlapping regions consistent with abundant convergent evolution among early tube dwelling taxa, suggesting that tube structure characters alone may not be sufficient to diagnose tubicolous animals to phylum level. The tubicolous Cambrian scalidophoran *Selkirkia* is a clear case study as this taxon shares no tube characters with extant tubicolous priapulids, is otherwise comparable to contemporary annulated tubicolous organisms based on our tube morphospace dataset (figure 4*c*,*d*) and it is unlikely that it would be correctly diagnosed to the correct phylum in the absence of soft tissue data.

None of the tubular taxa previously interpreted as annelids preserve characters (e.g. soft tissues) indicative of an annelid body plan, and the tube characters linking them to annelids are not sufficiently diagnostic to identify them as members of extant annelid subclades or their total group. Extant annelids that build tubes with extensive external annulation are restricted to a subset of Chaetopteridae, Siboglinidae, Maldanidae, certain serpulids and some species of Onuphidae. The majority of annelid tubes are irregular constructions from mucous, with or without agglutination of foreign particles. No unambiguous living or extinct annelids use calcium phosphate as a tube building material, while members of Sabellidae evolved tubes composed of calcium carbonate, which appeared in the late Palaeozoic and radiated mainly during the Mesozoic, with calcifying cirratulids originating much later, in the Cenozoic [44]. Calcium phosphate biomineralization in annelids appears to be restricted to the chaetae of Amphinomida [45], an errant group that is distantly related to the tubicolous taxa with which Cambrian tubes are compared [46,47].

Of the relevant fossil tube-building groups outlined above, the only groups that do not become extinct during or shortly after the Cambrian are those that are less ambiguously interpreted as cnidarians to the exclusion of annelids, i.e. conulariids [14], *Byronia* [29] and *Sphenothallus* [31,48] and there are gaps of hundreds of millions of years between these early tubicolous fossils and unambiguous records of tube-building annelid clades. This issue has been raised previously regarding interpretations of *Sabellidites* as a member of Siboglinidae, a clade whose less ambiguous fossil occurrences are far younger [21] and is inconsistent with the fossil record of crown group annelids, given their nested position within the Sedentaria [47], which along with its sister group (Errantia/Sedentaria) has no apparent fossil record before the Ordovician or maximally latest Cambrian [49]. Currently, the only known annelid tube dweller from the Cambrian is a member of Magelonidae [5], which lived in an organic (likely mucous) tube or lined burrow, sharing no features with the taxa under discussion here. Given the diversity of tubes that they produce, Annelida is frequently used as a wastebasket taxon into which poorly understood tubicolous taxa have been placed. This issue is exemplified by the case of Palaeozoic lophophorate ‘*Spirorbis*’ [50], which predates records of true serpulids by hundreds of millions of years, analogous to the comparisons made between late Ediacaran–Cambrian tubes and Siboglinidae.

The oldest records of tube dwelling cnidarians are found in the terminal Ediacaran, which include the oldest known conulariids from Brazil [51] and a stem group medusozoan preserving soft tissues that occurs alongside the classical Avalonian ‘Ediacara Biota’ [27] of Charnwood Forest. Some additional Ediacaran taxa have been discussed as possible cnidarians, primarily *Cloudina* and potentially related taxa that range into the Cambrian [10]. These taxa exhibit a variable tubular morphology and often exhibit a mode of skeletal growth in which apical deposition of shell laminae results in a morphology resembling stacked funnels. While recently the inferred presence of a discrete, internal gut [3] in cloudinids invited comparisons to bilaterian taxa (e.g. tubicolous annelids), such a tubular gut is not incompatible with the condition in elongate cnidarian polyps [27], such as that of *G. aspera.* A potential annelid affinity for *Cloudina* has previously been criticized due to lack of detailed similarities shared between *Cloudina* and calcareous annelids [12] and the annelid groups with which cloudiniids are currently compared (i.e. Siboglinidae) are unlikely to range into the early Palaeozoic [21]. More recently, a study proposed morphological links between *Cloudina* and unambiguous cnidarians from the Cambrian (*Cambroctoconus* and relatives), and suggested that *Cloudina* and its close relatives are a rare example of a clade that was diverse either side of the Ediacaran–Cambrian boundary [10].

Among extant cnidarians, an extensive, accretionary tubular exoskeleton is restricted to medusozoans, more specifically coronate scyphozoans [33]. However, tubicolous cnidarians identified from the Cambrian in particular deviate from living medusozoans in many respects, including body symmetry (tri-, hexa, penta radiality as compared to the canonical tetraradiate symmetry in the meduzoan crown group) and overall form. While some previous analyses have favoured placing this diversity of early tube dwelling cnidarians in the medusozoan crown, subtending or within Scyphozoa [4], more recent studies have placed them in the medusozoan stem lineage [27]. Our phylogenetic analysis places *Gangtoucunia* in a polytomy with living medusozoans, indicative of a position either within or proximal to the medusozoan crown group and the lack of resolution best attributed to the apparent character conflict between polypoid medusozoans and members of the Cambrian forms. Intriguingly, we do not recover a close relationship between *Gangtoucunia* in a clade with other medusozoans with calcium phosphate exoskeletons, suggesting that tube building materials could have a complex early evolutionary history, possibly due to convergent losses and reduction of calcium phosphate in skeletons as it became less available through the Palaeozoic [52]. The taxa identified as early cnidarians are also diverse in terms of skeletal ornament, transverse cross section and type of symmetry, indicating that early medusozoans were morphologically diverse. We, therefore, propose that early annulated tubular exoskeletons from the latest Ediacaran and Cambrian are better understood as variations on cnidarian exoskeletons rather than early annelids, in the absence of compelling and unambiguous soft tissue evidence.

## Data Availability

The data used in the tube morphospace analysis and phylogenetic analyses are available as part of the electronic supplementary material. The commands and topological constraints necessary to run the phylogenetic analyses are available as a separate MrBayes execuatable NEXUS file provided. The data are provided in electronic supplementary material [53].
